# Pandemical Influence on Athletic Events and Communications in Sport

**DOI:** 10.3389/fspor.2021.653291

**Published:** 2021-04-08

**Authors:** Victoria Zaborova, Konstantin Gurevich, Olga Chigirintseva, Victor Gavrilov, Vitaliy Rybakov

**Affiliations:** ^1^I.M. Sechenov First Moscow State Medical University, Moscow, Russia; ^2^Moscow Institute of Physics and Technology, Dolgoprudny, Russia; ^3^UNESCO Chair, Moscow State University of Medicine and Dentistry, Moscow, Russia

**Keywords:** coronavirus, epidemic, sports, online training, athletics

## Introduction

The first notifications of a new coronavirus infection came from China at the end of 2019 (The 2019-nCoV Outbreak Joint Field Epidemiology Investigation Team, 2020). In March 2020, WHO announced the onset of a new global pandemic [Coronavirus disease (COVID-19), 2019]. In Russia, the first cases of coronavirus infection were recorded in February of this year^1^. Currently, the epidemic has affected most countries of the world and more than half of the constituent entities of the Russian Federation.

Earlier than other countries, the epidemic began in China—and to date, most experts recognize it as liquidated (Arshad et al., 2020). At the same time, the main emphasis was placed on medical prophylactic, including isolation measures, as no specific treatment or vaccine against COVID-19 has been developed to date.

## Social Consequences of the Epidemic

Up till now there is no specific treatment for coronavirus infection, and a vaccine against it is only being developed, the main tactic of prevention is quarantine. The quarantine applies to persons who have been diagnosed with COVID-19, to those who have contacted them without special protective equipment, and also to people who have returned from epidemiologically disadvantaged regions.

The psychological effects of quarantine-related social exclusion over COVID-19 remains to be explored. However, the experience of previous epidemics indicates the development of depression, discomfort, loss of life values, and loss of confidence in the future. In some cases, psychological discomfort was the primary reason that people did not comply with the quarantine regime (Merchant and Lurie, 2020).

In addition, there is an overwhelming stream of information on the problem, including a lot of fake. The experience of previous epidemics shows that it is difficult for the population to navigate the flow of information, to choose reliable information from it. Some tend to panic (Shimizu, 2020). A number of studies have addressed the issue of emigration, stigma, racism, and COVID-19. It was noted that foreign students studying in China during the epidemic were most afraid of being separated from their families (Zhai and Du, 2020). Others were afraid that they would be humiliated at home as potential carriers of a new infection (British-Chinese people tell of ‘discrimination’ hate as fears rise over coronavirus, 2020).

People who were quarantined for COVID-19 showed an increased level of anxiety. This fact may be associated with frequently occurring sleep disorders in people who were in social isolation (Xiao et al., 2020a). In addition, sleep disturbances were noted in medical personnel who were forced to process during the epidemic and often faced a problem of misunderstanding on behalf of patients (Xiao et al., 2020b).

Another social consequence of quarantine associated with COVID-19 is the economic downturn (Kinross et al., 2020)^2^. It is caused by the fact that people who are in social isolation, to one degree or another, drop out of the real production sector. The consumer basket is changing. The demand for personal protective equipment and hygiene is growing. Health system costs are increasing (Ayittey et al., 2020). The economic impact of the COVID-19 epidemic globally remains to be assessed (Duan et al., 2020; Johnson et al., 2020). But now, a decline in tourism and the transport industry is already evident. How long such changes will last is not known.

## How the Epidemic Affects the Sports

One of the areas of life affected by the COVID-19 pandemic is sports. As part of the prevention of coronavirus transmission, mass sporting events are prohibited. Therefore, many competitions are either rescheduled or held without spectators (Table 1)^3^. NHL Cup discontinued ahead of time. Even the Olympic Games of 2020 were postponed (Gallego et al., 2020). Some international competitions are now held in virtual format. So, the FIFA-2020 championships of England, Spain, Italy will be held online^4^. Former Russian tennis player Marat Safin won the virtual US Open. In the final, the Russian defeated Spaniard Rafael Nadal. The winner was determined by voting on the official “twitter” and “Instagram” of the competition^5^.

**Table 1:** Examples of sporting events, the schedule of which has changed over the past 10 days due to coronavirus infection.

**Date**	**Competition**
25 March	DIVING: British Swimming cancels the London leg of the 2020 Fina Diving World Series. It had initially been postponed. FOOTBALL: The suspension of Japan's J League is extended into May, along with the associated cup competition. FOOTBALL: UNICEF's Soccer Aid fundraiser, scheduled to take place at Old Trafford on 6 June, is postponed. MOTORSPORT: The opening three rounds of the British Superbike Championship—at Silverstone, Oulton Park and Donington Park—are confirmed as postponed. SWIMMING: British Swimming cancels all its end of season domestic championships.
24 March	CYCLING: The fifth round of the UCI Mountain Bike World Cup, due to take place on 6–7 June in Fort William, is canceled. DARTS: All five Premier League events scheduled for April are postponed. DISABILITY SPORT: The Paralympic Games in Tokyo, scheduled to start on 25 August, are postponed until 2021. GOLF: Golf courses in England and Wales will shut following the latest government measures to tackle the coronavirus pandemic. HORSE RACING: Horse racing in Ireland is suspended until at least 19 April. OLYMPICS: The International Olympic Committee confirms Tokyo 2020, due to begin on 24 July, is postponed until next year. ROWING: The Henley Royal Regatta, which was due to be held from 1 to 5 July, is canceled. RUGBY UNION: The Champions Cup and Challenge Cup semi-finals and finals are postponed. Both finals were due to take place in Marseille on weekend of 22–23 May. DARTS: The fifth and sixth rounds of the world rally championship in Portugal and Italy, due to take place in May and June, are postponed
23 March	BOXING: British Boxing Board of Control suspends all events until the end of April. FOOTBALL: All football in Spain, including La Liga, is put on hold indefinitely while the country deals with the spread of coronavirus. FOOTBALL: The Irish Football Association extends the suspension of the football season in Northern Ireland until 30 April. FOOTBALL: Uefa postpones the Champions League and Europa League finals, scheduled for 30 May and 27 May respectively, as well as the Women's Champions League final, which was due to take place on 24 May. FORMULA 1: The Azerbaijan Grand Prix, due to take place on 7 June, becomes the eighth race of the 2020 season to be postponed or canceled. RUGBY LEAGUE: Australia's National Rugby League, which had been continuing behind closed doors, is suspended.
22 March	FOOTBALL: Former Manchester United midfeveryielder Marouane Fellaini, who plays for Chinese club Shandong Luneng, confirms he has tested positive for coronavirus. HORSE RACING: The Dubai World Cup, one of the world's richest horse races, due to take place on 28 March, is postponed. OLYMPICS: The International Olympic Committee gives itself a four-week deadline to make a decision on the postponement of Tokyo 2020.
21 March	CRICKET: The proposed seven-match series between Ireland and Bangladesh is postponed. The games were scheduled to be held in Belfast and England in May. FOOTBALL: Southampton chief executive Martin Semmens suggests the Premier League could resume before virus restrictions are lifted. FOOTBALL: Former Real Madrid president Lorenzo Sanz dies after being taken to hospital with coronavirus. FOOTBALL: Juventus and Argentina forward Paulo Dybala and former Italy captain Paolo Maldini test positive for coronavirus. FOOTBALL: Three Portsmouth players - James Bolton, Andy Cannon and Sean Raggett—test positive for coronavirus. FORMULA 1: World champion Lewis Hamilton says he is self-isolating after coming into contact with actor Idris Elba, who has tested positive for coronavirus. ICE HOCKEY: The International Ice Hockey Federation cancels the 2020 World Championship in Zurich and Lausanne. OLYMPICS: USA Track and Field, athletics' US governing body, calls for this summer's Olympics in Tokyo to be delayed. OLYMPICS: The Brazilian Olympic Committee (COB) calls for this year's Tokyo Olympics to be suspended.
20 March	AQUATICS: The European Aquatics Championships (swimming, diving, artistic (formerly synchronized) swimming and water polo) scheduled to take place in Budapest in May are postponed and provisionally rescheduled for 17–30 August. CRICKET: The ECB announces that the county cricket season is delayed by seven weeks and will not start before 28 May. RUGBY UNION: The Welsh Rugby Union has canceled all league and cup competitions for the rest of the season. The cancellation means there will be no promotion or relegation in any WRU league, with all teams remaining in their current division. RUGBY UNION: The RFU confirms the end of the 2019–2020 season for all levels below the Premiership because of coronavirus. SKATEBOARDING: The World Skate Olympic qualifying event in Long Beach, California, scheduled for 7–10 May, is postponed. SNOOKER: The World Snooker Championship, which was scheduled to take place from 18 April to 4 May, has been postponed.
19 March	DARTS: The Professional Darts Corporation announces the Players Championship double-headers and Unicorn Challenge Tour weekend planned during April have been postponed. FOOTBALL: English football is suspended until 30 April at the earliest, with the end of the 2019–2020 season extended indefinitely. FOOTBALL: Borussia Monchengladbach's players and coaching staff accept pay cuts during the coronavirus crisis. FOOTBALL: The Nigeria Football Federation (NFF) suspends its football league for four weeks due to the outbreak. FOOTBALL: Major League Soccer extends the suspension of all matches, with a target return date of 10 May. FOOTBALL: The Turkish Super Lig is suspended—the last major European league to do so. FOOTBALL: Rotherham manager Paul Warne confirms two of his players are in self-isolation after displaying symptoms of coronavirus. FORMULA 1: The Monaco Grand Prix is canceled and the Dutch and Spanish Grands Prix are postponed, with F1 bosses also announcing a delay to new regulations planned for 2021. HOCKEY: Great Britain's FIH Pro League games on 2–3 May and 16–17 May are postponed. MOTORSPORT: The Southern 100 road races due to take place in July on the Isle of Man are canceled. NFL: New Orleans Saints head coach Sean Payton tests positive for coronavirus, the first known case in the NFL.
18 March	ATHLETICS: All 675 UK Parkrun events are put on hold, initially until the end of March. BASEBALL: A Cincinnati Reds employee based at their spring training facility in Arizona has tested positive for coronavirus.
	CRICKET: The ECB recommends all forms of recreational cricket is suspended until further notice. FOOTBALL: The Asian Football Confederation announces all matches in the AFC Cup tournament, the second-tier club competition, are postponed. Games in the west of Asia had already been suspended. FOOTBALL: La Liga club Deportivo Alaves announce 15 staff members—including three players—have tested positive for coronavirus. FOOTBALL: The Leasing.com Trophy final between Portsmouth and Salford City on 5 April is postponed. FORMULA 1: The three-week summer break has been moved forward from August to March and April. GYMNASTICS: The Artistic Gymnastics All-Around World Cup event scheduled to take place in Tokyo in April is canceled. HORSE RACING: Racing in Ireland to continue but behind closed doors, with a maximum of one meeting per day. ICE HOCKEY: Great Britain's two fixtures against Hungary on 21 and 22 April are canceled. MOTORSPORT: The Le Mans 24 H, scheduled to take place on 13–14 June, has been postponed and rearranged for 19–20 September. MOTORSPORT: The IndyCar Grand Prix of Long Beach, scheduled for 19 April, is canceled. The race will return next year. TENNIS: ATP and WTA extend suspension of all top-level tennis until 7 June. TRIATHLON: British Triathlon announces the Leeds Triathlon, due to take place on 6-7- June, is postponed.
17 March	ATHLETICS: Diamond League postpones the first three meetings of the 2020 season, scheduled for Qatar and China. ATHLETICS: Club training sessions, events, competitions, club committee and face-to-face meetings, athlete camps, running groups and social events are suspended in England, Scotland and Wales. BASKETBALL: The British Basketball League postpones the 2019/20 season until further notice. BOXING: All boxing events canceled until the start of April by the British Boxing Board of Control. CRICKET: England cricketer Alex Hales is in self-isolation after developing coronavirus symptoms following his return to the UK from playing in the Pakistan Super League. CRICKET: Pakistan Super League is postponed, on the day the semi-finals were due to take place. CRICKET: ECB to meet on Thursday to look at possible rescheduling the cricket summer. The County Championship season is scheduled to start on 12 April, but counties are due to play fixtures from 2 April in preparation. CYCLING: The Tour de Yorkshire, scheduled to take place between 30 April and 3 May, is postponed with no new date yet announced. CYCLING: The sport's governing body, the UCI, postponed the prestigious one-day races Paris-Roubaix and Liege-Bastogne-Liege. Paris-Roubaix was due to be held on 12 April, with Liege-Bastogne-Liege two weeks later. The Fleche Wallone—one of the Ardennes classics—set for 22 April was also canceled. DARTS: The forthcoming PDC Development Tour events on 21–22 March and the Players Championship weekend on 28–29 March are postponed. DIVING: The Fina Diving World Series finale, which was scheduled to take place at the London Aquatics Center between 27 and 29 March, is postponed. EQUESTRIAN: British Eventing cancels all fixtures with immediate effect, a fortnight after its scheduled eight-month season began. FOOTBALL: Euro 2020 has been postponed until 2021. FOOTBALL: Liverpool announce the final Hillsborough memorial service, due to take place at Anfield on 15 April, is postponed. FOOTBALL: The two-legged play-off between Cameroon and Chile for a place at the 2020 Tokyo Olympics, initially scheduled for April, is postponed. FOOTBALL: The African Nations Championship, for players who are based in Africa, is postponed. GYMNASTICS: The women's European Championships set for the end of April in Paris and the men's event in Baku at the end of May are canceled by the European Gymnastics Union. HORSE RACING: All racing in Great Britain will be suspended from Wednesday until the end of April. HORSE RACING: The 146th Kentucky Derby in the United States will be rescheduled from 2 May to 5 September. MIXED MARTIAL ARTS: UFC president Dana White says the next three fight nights are postponed. SNOOKER: World Snooker postpones the Coral Tour Championship in Llandudno, which was set to begin on Tuesday. SURFING: World Surf League cancels or postpones all events until at least the end of May. SWIMMING: British Swimming announces the GB Olympic and Paralympic Trials, due to take place between 14 and 19 April are canceled. TENNIS: French Open postponed until September. TRAMPOLINING: The European Trampoline Championships in Sweden from 7 to 10 May are called off. TRIATHLON: British Triathlon, Triathlon England, Triathlon Scotland and Welsh Triathlon suspend organized triathlon activity until at least 30 April.

In many countries, as part of quarantine measures, sports sections and circles have stopped working. Due to social isolation, people reduce their daily activity, which can lead to an increased likelihood of developing and/or progressing chronic non-communicable diseases. Special complexes of physical exercises are being developed for people forced to quarantine (Chen et al., 2020).

A number of famous athletes were infected with a coronavirus, for example, Thiago Ceibut-Wild, Marco Sportiello, trainer Fatih Terim. Boxer Anthony Joshua quit self-isolation since he was in contact with Prince Chals. Lionel Messi, Cristiano Ronaldo, Conor McGregor, Georges Mendes and several other famous athletes donated money for the treatment of patients with COVID-19. Such messages caused a great resonance in the media and attracted increased attention to the topics of sports and COVID-19. Due to the economic downturn, spending on sports is declining. So Madrid REAL will soon train without wages.

## Change in Sports Communication Due to Coronavirus

In many countries of the world, a self-isolation regime has been introduced, which suggests that the movement of people outside their own apartment (house) is minimized. Often during the quarantine period, a ban on visiting sports infrastructure facilities is introduced. All this makes it impossible to conduct training of sports teams. Therefore, some experts propose the development of special complexes for team members who are forced to stay at home in an epidemic.

A long restriction in the organization of the training process can lead to a local decline in results in all sports disciplines. It becomes extremely important to develop modern and effective training techniques in the face of such restrictions. The sports world has not encountered a similar situation before and this is a huge challenge for the entire sports scientific community. At the same time, this can become an impetus for the revision of views on the existing approaches in the training of athletes, the development and implementation of completely new views on the construction of the training process, which will remain effective in sports after the end of restrictive measures.

For example, Playmaker School organized online basketball lessons for children at home. The only thing is that you need to have high ceilings (at least 3.5 m), while the standard height of the ceilings of apartments in Russia is 2.5 m.

Irina Wiener International Sports Academy has organized a virtual sports hall for students. Children will learn how to perform basic gymnastic exercises at home using telecommunication technologies. Video lessons are grouped by the age categories of students: a set of exercises for preschoolers is designed to form the correct posture and musculoskeletal system, as well as to develop flexibility, and the number of exercises for primary school students includes physical education, dance exercises, exercise^6^. The most difficult types of sports are those where a stadium or sports ground is usually required for training. For example, Juventus Academia has developed a special set of exercises that can be performed at home, but at the same time carry out training on the speed of reaction, running, passing the ball. It is proposed to make a video from your own training, send it to receive feedback.

Another football school for adults Life & football has developed temporarily suspended training. The system of exercises is currently being reviewed and a quarantine strategy is being developed without going online, as school representatives consider it impossible to train without the personal interaction of team members^7^. Due to social isolation, people reduce their daily activity, which can lead to an increased likelihood of developing and/or progressing chronic noncommunicable diseases. A number of researchers emphasize the importance of maintaining high physical activity even in an epidemic. This is a factor in the prevention of a number of chronic noncommunicable diseases. In addition, physical activity allows you to maintain a positive mood and prevents the development of depression. However, for most people, the training conditions need to be reviewed, due to the limited dimensions of their own housing, the lack of special equipment or its insufficient completeness, etc. Special complexes of physical exercises are being developed for people forced to quarantine^8^. The All-Russian Physical Culture and Sports Society “Labor Reserves” joined the action of the Ministry of Sports of the Russian Federation “Train at home. Sport is the norm.” As part of the Promotion, the Company developed a free training marathon so that the Russians did not stop engaging in physical activity for the time of self-isolation. Anyone can take part in it, regardless of age and geography. All exercises can be performed at home, without violating the regime of self-isolation. More than 2,500 participants have already registered for the first stream of free training, and not only from Russia. In online mode, they train with society from Ukraine, Belarus, Germany, and Spain. More than 2.5 thousand participants have already registered for the second stream. In many countries, as part of quarantine measures, sports sections and circles have stopped working. On-line courses are offered for persons who wish to continue sports with a trainer in the Russian Federation. So, Taekwondo Kerugi Schools arrange classes through Zoom. Fight Pro Unity Club uses the Ivideon service^9^. Fitness clubs in Moscow UFC Gym network launched many directions for online training in the YouTube format^10^. Similar programs are at the fitness clubs World Class, SM Stretching and others. Large manufacturers of sportswear, such as Adidas, Nike, have also launched on-line fitness classes. A large number of fitness video lessons are available free of charge for quarantine events on Russian online television Okko.

## Discussion

At the moment, the entire world sports industry has “stopped.” Thus, the structural scheme of the functioning of all world sport, which has developed over the years, is being violated. And the longer the restrictions last, the more difficult it will be to restore the system. Each sport has years of competition calendars and training schedules. Since many sports have seasonal features, the progress of the competition is possible either for a short period or for a whole cycle for a year.

To date, all summer sports have already fallen into the risk category. However, there is also a larger four-year cycle of sports events tied to the Olympics, from which everything is built up and the shift of any tournament by one year will make serious adjustments for the next years. Each leap year there is a summer Olympic Games, which is the main start of the four-year period, and accordingly for 3 years various continental and world championships are distributed between the Olympic Games, the fate of which is now in question. All continental championships and the Olympics planned for 2020 have been moved forward a year, which immediately raises a number of questions about what will happen to the major tournaments planned in 2021 and beyond, since it is impossible to hold tournaments in one season at once in two years. Thus, it can be assumed that with the long-term global restrictions associated with the pandemic, some of the tournaments will simply be canceled, which will be a serious blow to both the financial and ethical aspects of the sport. All sporting events have their so-called “rank,” and each competition, depending on their level, is a developmental stage for athletes personally and for teams in general. Separately, it is worth noting that competitions have age restrictions for participants, various championships among juniors and youths. At this age, every start is worth its weight in gold for the development of an athlete. At the moment, many continental and world championships among juniors and youths have already been canceled (postponed indefinitely). Most likely, they can be canceled and a whole generation of young athletes will be left without the most important stage of sports development. In such a situation, it will become especially important to develop measures to support young athletes, preserve motivation to continue their sports career.

At present, cases of the disease have been recorded in more than two hundred countries of the world, the isolation regime has covered about four billion people on the planet, the vast majority of sporting events have been stopped and measures aimed at combating the spread of COVID-19 are only being tightened. The global sports industry will have to go through a difficult path of “isolation” and further recovery. The further impact of the COVID-19 epidemic on athletic life and communication in sports has yet to be evaluated.

## Author Contributions

KG: idea and designing. VZ: data analysis. OC: literature overview. VG: sport analysis. VR: analysis of online sports. All authors contributed to the article and approved the submitted version.

## Conflict of Interest

The authors declare that the research was conducted in the absence of any commercial or financial relationships that could be construed as a potential conflict of interest.

## Footnotes

^1^COVID-19 coronavirus pandemic. Retrieved from https://www.worldometers.info/coronavirus/#countries.html.

^2^The impact of the pandemic on people and society. Retrieved from http://www.ras.ru/News/ShowNews.aspx?ID=27f8355f-42dd-4bee-9f7a-6b1ea8602e2d.

^3^Coronavirus: How the virus has impacted sporting events around the world. Retrieved from https://www.bbc.com/sport/51605235.html.

^4^Football cannot be stopped! Championship launches FIFA20 online tournament. Retrieved from https://www.championat.com/football/article-3999419-chempionat-zapuskaet-onlajn-turnir-po-fifa20.html.

^5^Safin won the virtual US Open. Retrieved from https://www.championat.com/tennis/news-4012101-safin-stal-pobeditelem-virtualnogo-us-open.html.

^6^Gymnastics online: for students opened a virtual gym. Retrieved from https://www.championat.com/other/news-4012027-gimnastika-onlajn-dlja-shkolnikov-otkryli-virtualnyj-sportivnyj-zal.html.

^7^Football school for adults: survive at all costs. Retrieved from http://vc.ru/offline/116810-futbolnaya-shkola-dlya-vzroslyh-vyzhit-lyuboy-cenoy.

^8^Labor Reserves Society launched an all-Russian online project of free training. Retrieved from https://trudovyerezervy.ru/news/obshchestvo-trudovye-rezervyzapustilo-vserossiyskiy-onlayn-proekt-besplatnykh-trenirovok/html.

^9^Musculoskeletal system formation. Retrieved from http://academyviner.com/news_post/news_post/formirovanie-kostno-myshechnoj-sistemy.

^10^There is a way out. Where to train online for free. Retrieved from https://www.championat.com/lifestyle/article-4003583.
